# Reconstructing biological molecules with help from video gamers

**DOI:** 10.1107/S2059798325008149

**Published:** 2025-10-08

**Authors:** Andreas C. Petrides, Robbie P. Joosten, Foldit Players, Firas Khatib, Scott Horowitz

**Affiliations:** a Department of Computer and Information Science University of Massachusetts Dartmouth Dartmouth Massachusetts USA; b Oncode Institute and Division of Biochemistry Netherlands Cancer Institute Plesmanlaan 121 1066 CXAmsterdam The Netherlands; c Department of Chemistry and Biochemistry and The Knoebel Institute for Healthy Aging University of Denver Denver Colorado USA; d University of Denver, USA; Lawrence Berkeley National Laboratory, USA

**Keywords:** *Foldit*, *PDB-REDO*, citizen science, model curation

## Abstract

*Foldit* is a citizen science video game in which players tackle a variety of complex biochemistry puzzles. Here, we describe a new series of puzzles in which *Foldit* players improve the accuracy of models in the public repository of experimental protein structure models, the Protein Data Bank (PDB). Analyzing the results of these puzzles showed that the *Foldit* players were able to considerably improve the deposited structures. We describe a mechanism by which the efforts of the *Foldit* players can be fed back into the structural biology scientific record by using *Foldit* results as improved input for the PDB-REDO databank. These efforts highlight the continued need for the engagement of the lay population in science.

## Abstract 2

The results of the *Foldit* citizen science puzzles for crystallographic models and their integration into the PDB-REDO databank are discussed.

## 1. Introduction

Our shared knowledge of experimental biomolecular structures is stored in the Protein Data Bank (PDB; Burley *et al.*, 2019 ▸). The accuracy of the models in the PDB is important for multiple reasons. For example, the recent successes in deep-learning approaches to predicting protein structure (Jumper *et al.*, 2021 ▸) rely on an accurate PDB for continued development. Large sets of PDB entries are used to study specific aspects of macromolecular structure such as side-chain conformations (Lovell *et al.*, 2000 ▸), Ramachandran plot distributions (Sobolev *et al.*, 2020 ▸), hydrogen bonding (van Beusekom, Touw *et al.*, 2018 ▸), metal-binding sites (Zheng *et al.*, 2017 ▸; Putignano *et al.*, 2018 ▸), nucleic acid conformations (de Vries *et al.*, 2021 ▸; Schneider *et al.*, 2018 ▸) and post-translational modifications (Schofield *et al.*, 2024 ▸). Importantly, the PDB serves as a resource for biologists and biochemists to develop hypotheses to test in their everyday experiments. Mistakes in structure models can cause scientists to base their hypotheses on incorrect data, which causes delays in scientific progress.

The PDB contains many solvable errors at different levels of complexity (Joosten *et al.*, 2012 ▸). The data used in crystallography and cryo-EM – maps of electron density or electron potential, respectively – have limitations as they are the result of an indirect experiment with experimental error and limited resolution. Peaks in the distribution of electron density or electron potential represent atomic positions; thus, scientists can fit atoms to these maps to discover the atomic structure of the macromolecule in the experiment. This requires a combined approach of visual fitting of these maps as well as computational tools to aid in complying with the known rules of chemistry and physics in how molecules are put together. Limitations include low resolution of the data, parts of the maps where artifacts or missing data can cause blurriness in the maps and assumptions in the computational models used to process the primary experimental data, among other causes of low data quality. As a result, the scientists interpreting these data can make mistakes. High attention to detail and the use of verification tools can help to prevent some mistakes (Read *et al.*, 2011 ▸), but the sheer quantity of data makes it likely that human error will still persist into published and deposited structure models. Furthermore, the PDB does not require peer review for deposited models and as a result many entries contain errors, ranging from inconsequential to egregious.

For this reason, the *PDB-REDO* project was begun in 2006 (Joosten & Vriend, 2007 ▸). The mission of *PDB-REDO* is to perform automated re-refinement and rebuilding of the crystallographic structure models in the PDB to improve the accuracy of PDB entries and remove model errors in the process. This venture has been successful, with the PDB-REDO databank now containing over 180 000 entries, many of which have an improved fit to the experimental data and more probable structural features (van Beusekom, Touw *et al.*, 2018 ▸).

However, is there still a need for improvement of structure models beyond the automated re-refinement by *PDB-REDO*? Yes, the procedure in *PDB-REDO* looks at many structural aspects, but it is limited to model issues that can be handled robustly with a very low risk of making the model worse. Indeed, a search of the PDB for its lowest quality structure models shows that the improvements by *PDB-REDO* in these cases can be modest at best. They still appear to be relatively low quality after *PDB-REDO* refinement. This suggests that new approaches are needed.

One possible approach is to enlist the aid of humans to improve the PDB via the players of the biochemistry video game *Foldit. Foldit* is a citizen science game in which players work on a variety of complex biochemistry puzzles, collaborating and competing to create the best possible structure model (Cooper *et al.*, 2010 ▸). Model quality within the game is judged by the Rosetta force field (Leman *et al.*, 2020 ▸), combined with other elements, such as the fit to an underlying map from experimental data (Horowitz *et al.*, 2016 ▸). Previous competitions have shown that *Foldit* players can solve both crystal structures and cryo-EM structures with higher accuracy than scientists or computational algorithms. The *Foldit* players were especially adept at improving Ramachandran space usage and reducing steric clashes while not reducing the fit to data (Horowitz *et al.*, 2016 ▸; Khatib *et al.*, 2019 ▸). Therefore, it became an obvious question to ask whether *Foldit* players could also improve the structures already within the PDB and whether this could improve the performance of *PDB-REDO* in cases with very low starting quality.

To test this possibility, we created a new series of *Foldit* puzzles, termed *Reconstruction puzzles*. In these puzzles, *Foldit* players were given protein structure models and the underlying density maps and tasked with improving the structures. We then tested whether the puzzle solutions outperformed the underlying PDB models as input data to *PDB-REDO*. In this report, we discuss the results after the first 58 of these puzzles. We also discuss our newly created workflow that automatically connects new puzzle solutions to the PDB-REDO databank.

## 2. Methods

### 2.1. Data-set selection

To test whether existing PDB entries could be improved by *Foldit* players, we selected 58 entries solved by X-ray crystallography with available experimental data. The structures were chosen to primarily be protein-only and to have poor model and/or data-fit parameters. Most structures were chosen by hand using PDBe quality metrics. Primarily, models with poor Ramachandran plots and many steric clashes were chosen within this data set. The models have resolutions ranging from 1.3 to 3.5 Å, were deposited from 1988 to 2022, and contained between 499 and 10 052 atoms.

### 2.2. *Reconstruction puzzle* setup

*Reconstruction puzzles* were prepared by performing five rounds of refinement, including simulated annealing, in *Phenix* (Liebschner *et al.*, 2019 ▸) using the original PDB entry with all ligands and waters removed. This new model was given to the players with a feature-enhanced map (Afonine *et al.*, 2015 ▸) for reconstruction within the *Foldit* application. The tools available include both local and global minimization, distance constraints and interactive chain movement. The puzzles were made available sequentially, with each staying available to users for seven days.

### 2.3. Puzzle-solution processing

A set of post-processing steps occurred following the completion of each puzzle.

A Python script was used to orchestrate the clustering of player-generated solution files based on their structural similarity, employing the C^α^ root-mean-square deviation (CA-RMSD) metric, starting with the top-scoring solution for that *Foldit* puzzle. The score for a puzzle solution is judged by the Rosetta force field (Leman *et al.*, 2020 ▸) and the fit to the underlying map from experimental data (Horowitz *et al.*, 2016 ▸). Iteratively, the script varies the threshold for CA-RMSD clustering, starting from 1 Å, searching for the next-highest-scoring solution that is at least 1 Å CA-RMSD away, and decreasing that threshold until the total number of distinct clusters is greater than 100. So far, the *Foldit* community have generated up to 150 000 solutions for a single *Reconstruction puzzle*. Therefore, this iterative clustering process is crucial to reduce the results to a concise set of models.

Following clustering, the solutions were clustered and ranked based on their *Foldit* score, with the top 100 clustered solutions moving to the next step. The Python script then submitted the top 100 clustered solutions by score to the API of the *PDB-REDO* webserver (Joosten *et al.*, 2014 ▸), facilitating the submission of these clustered solutions for further refinement and analysis.

The *PDB-REDO* procedure used here entailed optimizing the weight between the experimental data (as retrieved from the PDB) and restraints for covalent geometry and atomic *B* factors to maximize the fit to the experimental data whilst maintaining normal geometry. Combinations of different types of additional restraints were used while refining the models with *REFMAC* (Kovalevskiy *et al.*, 2018 ▸): homology-based hydrogen-bond restraints (van Beusekom, Touw *et al.*, 2018 ▸) were applied with eight solution sets, general hydrogen-bond restraints with 18 sets, local noncrystallographic symmetry restraints with 26 sets and jelly-body restraints with 19 sets (Murshudov *et al.*, 2011 ▸). All of the model-rebuilding steps in *PDB-REDO* were excluded to stay close to the original solution.

Upon completion of the *PDB-REDO* calculations, the finalized structure models and associated metrics were retrieved. The top 10 (by *R*_free_) redone models by were written into new PDB files and the corresponding refinement statistics were organized into comprehensive data dictionaries and graphs, both of which facilitate further analysis. A specialized *picker* algorithm (Joosten *et al.*, 2012 ▸) then performed the final model selection by first rejecting all candidates that had bond-length or bond-angle r.m.s.*Z* values greater than 1.0. Models that showed clear signs of overfitting based on the ratio between *R*_free_ and the *R* factor were also rejected (Joosten *et al.*, 2012 ▸). The remaining models were sorted by *R*_free_ and the model with the lowest value was selected as the winning model.

### 2.4. Testing in *PDB-REDO*

For each PDB entry in the data set two full *PDB-REDO* calculations were run (including model rebuilding), one with the original PDB model as input and one with the top puzzle solution as input. The resulting models were analyzed with *WHAT_CHECK* (Hooft *et al.*, 1996 ▸), *Tortoize* (van Beusekom, Joosten *et al.*, 2018 ▸) and *MolProbity* (Williams *et al.*, 2018 ▸).

## 3. Results and discussion

### 3.1. *Reconstruction puzzle* results

In total 58 *Foldit* puzzles were performed. On average, each puzzle had ∼80 000 submitted solutions. After clustering and scoring, the top cluster solutions were submitted to the *PDB-REDO* server to see how these performed in reciprocal-space refinement. A winning solution was selected based on the algorithm in Section 2.3.

How did the *Foldit* players improve on these structures? To get an idea, we asked several *Foldit* players whose structure models were chosen as the best overall structure. The approaches taken by different players were quite variable (see Supplementary Information S1). In most cases, the strategy involved a combination of hand-fitting (interactively moving atoms in the graphical interface) as well as automated tools and scripts. However, in certain cases the top solutions used only scripts and automated tools, suggesting that considerable improvement in automated fitting of structural maps can be accomplished (Supplementary Information S1.2). The top player, Galaxie, improved ten out of 58 puzzles with the highest score. An analysis of their strategy is given in Supplementary Information S1.3.

### 3.2. *PDB-REDO* analysis and implementation

**Figure 1:**
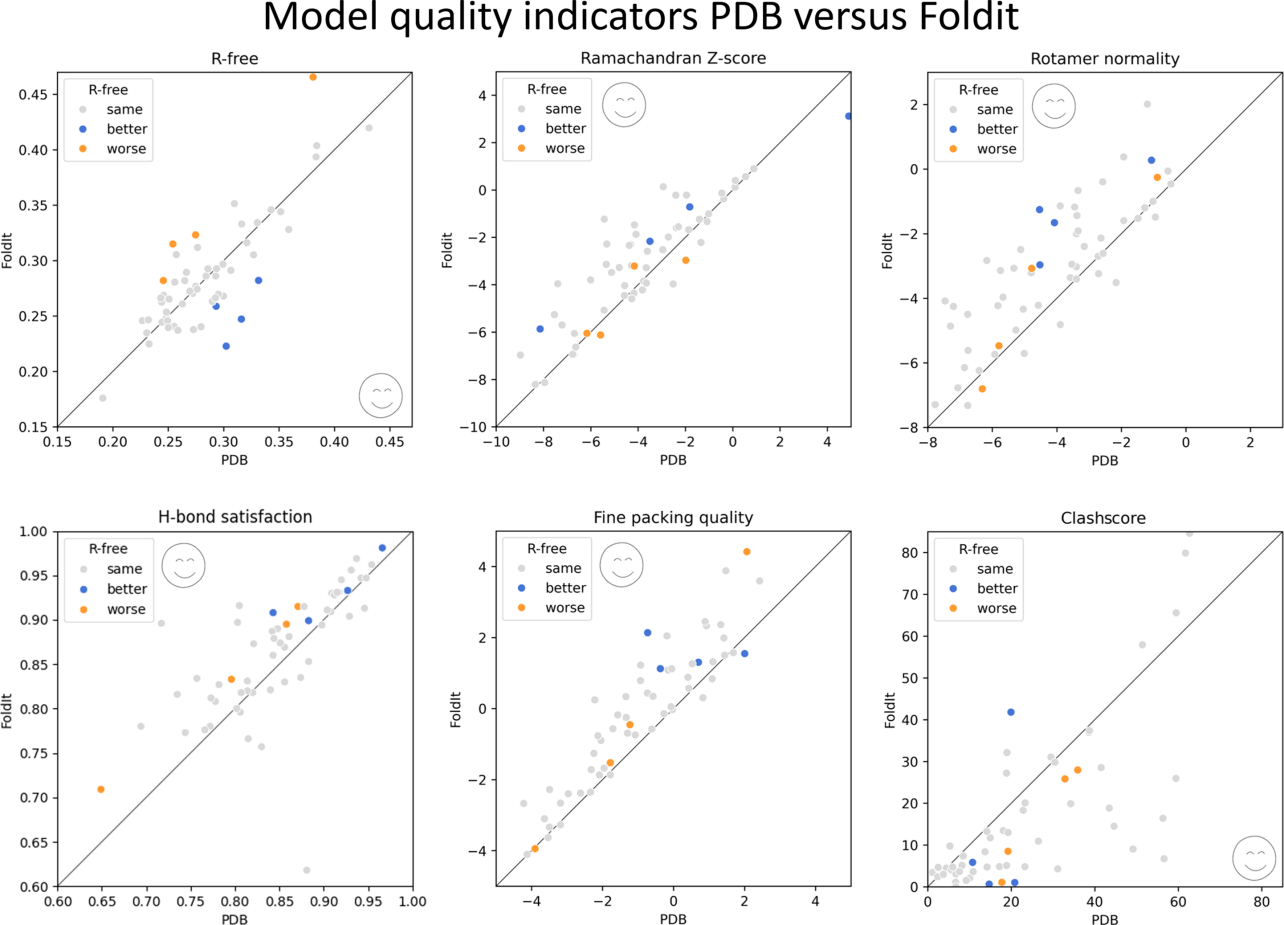
Comparison of model quality scores for *PDB-REDO* output using the original PDB model (*x* axis) or the *Foldit*-refined model (*y* axis) as input. Each point is a separate PDB entry. From top left to bottom right: *R*_free_ (from *REFMAC*), Ramachandran *Z*-score (from *Tortoize*), side-chain rotamer normality *Z*-score (from *Tortoize*), hydrogen-bond satisfaction fraction (from *WHAT_CHECK*), fine packing quality *Z*-score (from *WHAT_CHECK*) and clashscore (from *MolProbity*). Emoticons mark the side of the diagonal that indicates an improvement. Cases with a significant change in *R*_free_ when switching to the *Foldit* model are highlighted in each plot.

The winning solution of each puzzle was then run through the complete *PDB-REDO* pipeline for comparative analysis to see whether using the *Foldit* structure models as a starting point is an improvement over using the structure models from the PDB itself. The distribution of model quality metrics was plotted (Fig. 1 ▸). In 43 of the 58 cases, the overall structure quality (based on the overall *MolProbity* score) was improved by using the *Foldit* structure as the starting point. In general, the *Foldit* player structures had considerably improved chemical and physical properties in the vast majority of puzzles by multiple metrics (Fig. 1 ▸ and Supplementary Information S2). As seen by the *MolProbity* clashscore, the atomic clashes of the structure models were especially improved, but not in all cases. In terms of *R*_free_, there was no clear trend towards improvement or deterioration. Eight cases showed a significant change based on the criteria we previously established (de Vries *et al.*, 2021 ▸): four cases improved and four deteriorated.

To avoid adding cases to the PDB-REDO databank where the *Foldit* puzzle solution performs worse in terms of fit to the experimental data than the original PDB model, a model-selection algorithm had to be created that was sufficiently selective but, for efficiency, did not require running *PDB-REDO* twice. Many decisions in *PDB-REDO* are based on comparing refinements in which only one parameter (for example a restraint weight or the *B*-factor model) was changed (Joosten *et al.*, 2012 ▸), and we tested whether such a solution was possible in this case. After the initial calculation of *R* factors for the PDB model, as is the normal procedure in *PDB-REDO*, both the original PDB model and the *Foldit* solution are subjected to 20 cycles of restrained refinement in *REFMAC* with automatic weighting and isotropic *B* factors. Noncrystallographic symmetry and twinning are considered if required. Because *Foldit* puzzles are created only for PDB entries with very poor geometric quality and the *Foldit* scoring function promotes strong geometric improvement, it is assumed that the geometric quality of *Foldit* models is generally better than that of the PDB model. The best refinement result could therefore be selected with the *picker* program (Joosten *et al.*, 2012 ▸) that focuses on the fit to the experimental data. The *Foldit* model was selected as the best input model unless (i) the PDB model gave a free log likelihood (as reported for the *REFMAC* refinement) that was 6.7 points better than the *Foldit* model, which corresponds to a ‘decisive’ Bayes factor (Kass & Raftery, 1995 ▸), and (ii) *R*_free_ for the PDB model was at least σ*R*_free_, *i.e.* one estimated standard deviation of *R*_free_, lower (with σ*R*_free_ equal to *R*_free_ divided by the square root of the number of test-set reflections). This approach leaves the option of accepting a small deterioration in *R*_free_ to obtain (much) better geometric quality.

**Figure 2:**
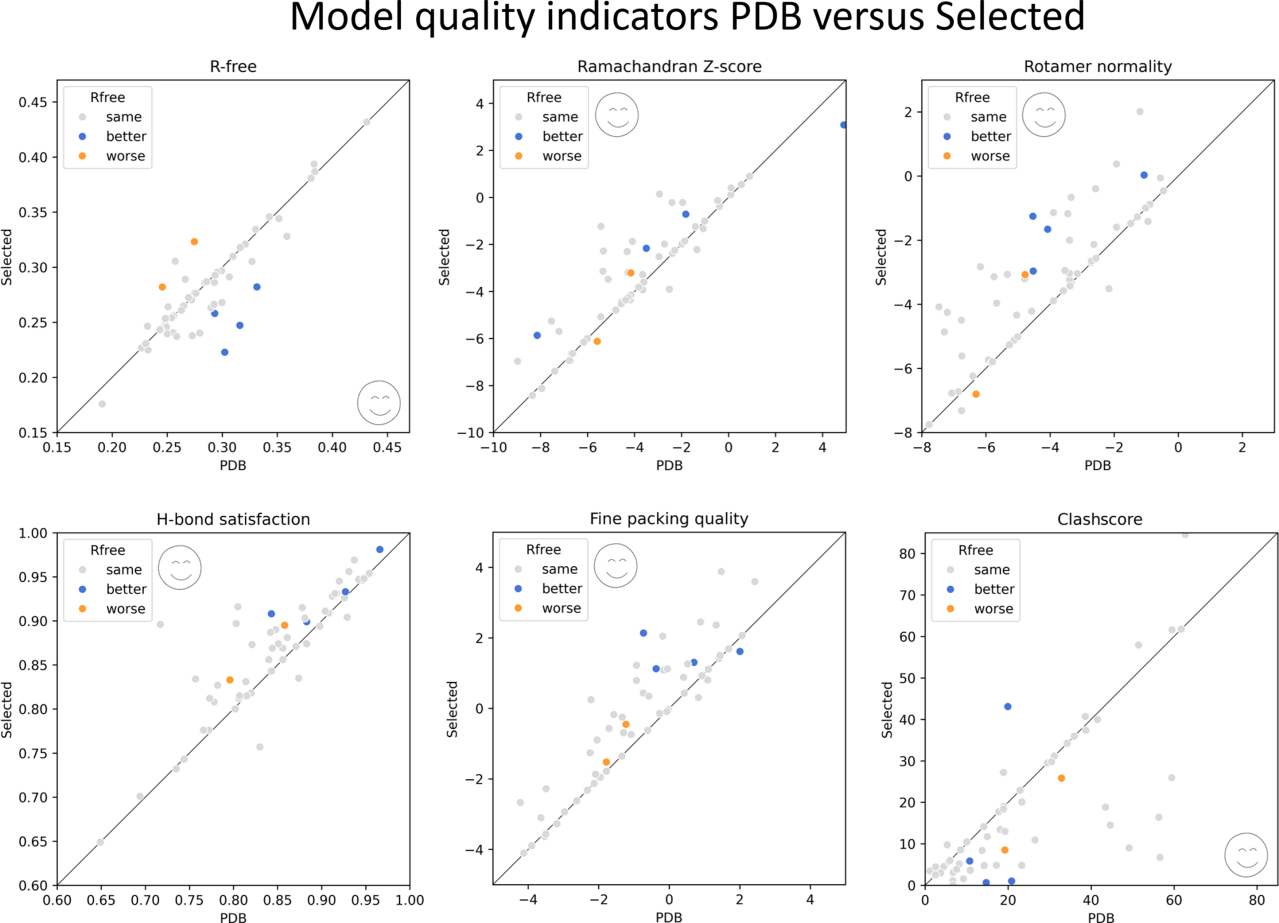
Comparison of model-quality scores for *PDB-REDO* output using the original PDB model (*x* axis) or the model selected by *picker* in *PDB-REDO* (*y* axis) as input. Each point is a separate PDB entry. From top left to bottom right: *R*_free_ (from *REFMAC*), Ramachandran *Z*-score (from *Tortoize*), side-chain rotamer normality *Z*-score (from *Tortoize*), hydrogen-bond satisfaction fraction (from *WHAT_CHECK*), fine packing quality *Z*-score (from *WHAT_CHECK*) and clashscore (from *MolProbity*). Emoticons mark the side of the diagonal that indicates an improvement. Cases with a significant change in *R*_free_ when switching to the *Foldit* model are highlighted in each plot.

A new *PDB-REDO* pipeline was created that, for each *PDB-REDO* run performed on a PDB entry, queries the *Foldit* database for the existence of a *Reconstruction puzzle* model. If this exists, the above test was performed. Of the 58 test cases, the *Foldit* model was selected 42 times (Supplementary Information S2). With respect to always using the *Foldit* model, this reduced the number of cases of model deterioration in terms of fit to the experimental data (*R*_free_) from 29 to 21, with an acceptable loss of geometric quality improvement (Fig. 2 ▸). For instance, for the overall *MolProbity* score improvement, the number of cases that improved was reduced from 43 to 33 and the number of cases that deteriorated was reduced from 13 to 10. Other geometric quality indicators followed the same trend (Supplementary Information S2).

### 3.3. Yeast mitochondrial import inner membrane translocase subunit TIM44p

**Figure 3:**
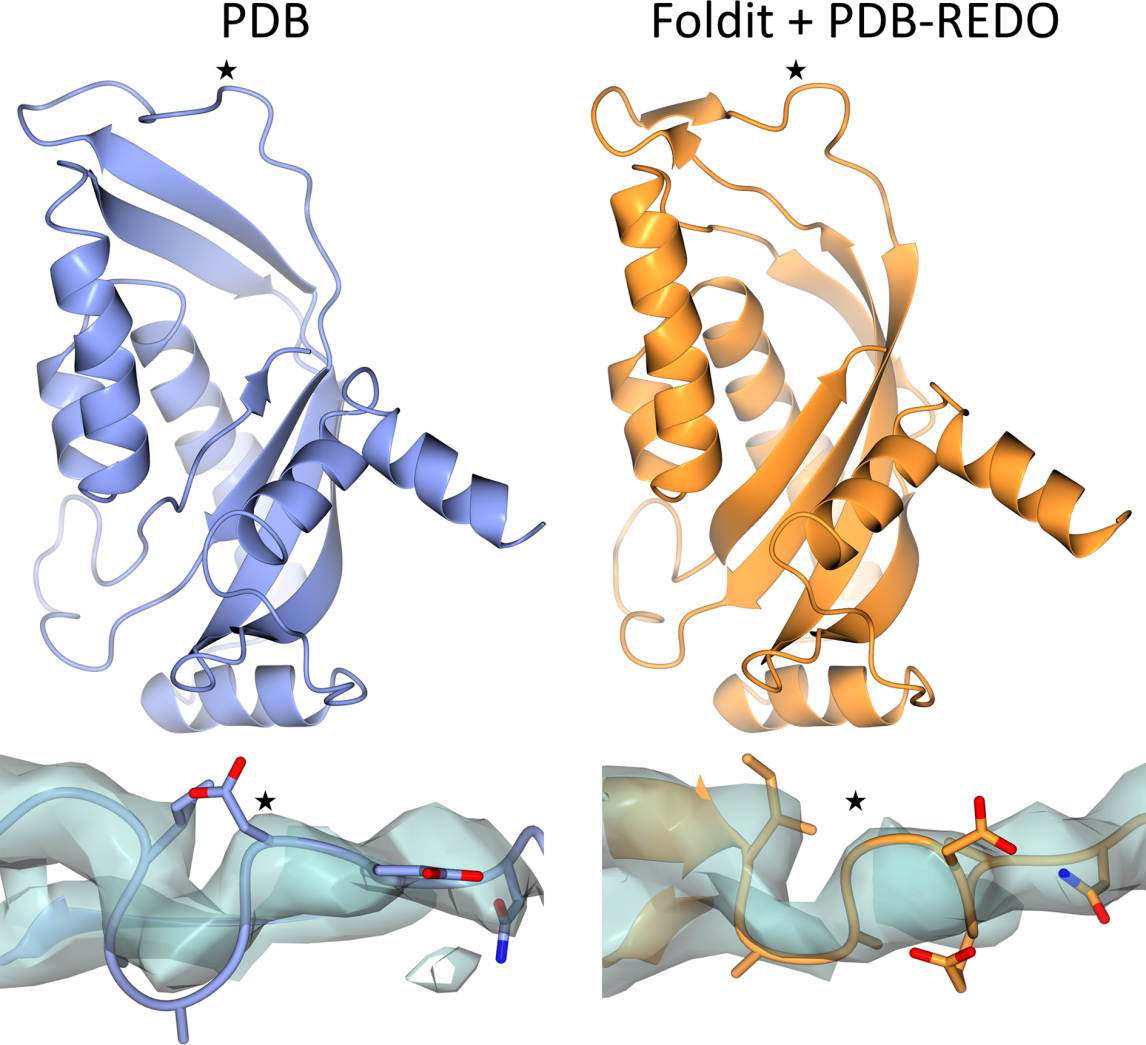
Comparison of the original PDB model (left) of yeast mitochondrial import inner membrane translocase subunit TIM44p (PDB entry 2fxt; Josyula *et al.*, 2006 ▸) with the *Foldit* model after* PDB-REDO* (right). Top: in the overall structure a clear change is visible, notably in the distribution of β-strands. The number of β-strand/β-bridge residues increases from 48 to 56. Bottom: rearrangement of a surface loop causes a register shift. At the position marked with a star there was originally an Asp residue, whereas in the updated model there is the fully conserved Gly388 residue. Models are shown with their corresponding 2*mF*_o_ − *DF*_c_ map contoured at 1.1σ. This figure was made with *CCP*4*mg* (McNicholas *et al.*, 2011 ▸).

There are cases in our test set where changes to the structure can be observed at many points. PDB entry 2fxt (Josyula *et al.*, 2006 ▸), the C-terminal domain of yeast mitochondrial import inner membrane translocase subunit TIM44p, which was solved at 3.2 Å resolution, is such a case. When comparing the original PDB model with the model made with *Foldit* followed by *PDB-REDO*, a clear change in secondary structure is observed, notably in the central β-sheet (Fig. 3 ▸). Analysis with *DSSP* (Hekkelman *et al.*, 2025 ▸) showed that the PDB model had 48 β-strand/β-bridge residues, whereas the updated model had 56. Overall, 121 out of 192 residues had an α/π-helical or β-strand/β-bridge secondary structure. In the updated model this was 134 residues out of 192.

In many of the cases though the changes are localized, and often in regions of poor electron density such as loops. However, these regions can be important biologically. In PDB entry 2fxt we observe that Gly388 has changed position by 3.2 Å due to a so-called register shift (Fig. 3 ▸). This glycine is fully conserved over 1522 sequences in *HSSP* (Joosten *et al.*, 2011 ▸) and this glycine is also conserved in the more distant human ortholog (UniProt O43615). Moreover, *AlphaMissense* (Cheng *et al.*, 2023 ▸) suggests that mutations to this residue are very likely to be pathogenic (minimal score 0.922), indicating that this a key residue in the protein.

### 3.4. Availability in the PDB-REDO databank

With the success of the *Reconstruction puzzle* series, we have created a new pipeline in which data from *Foldit* puzzles can be automatically analyzed and evaluated by *PDB-REDO* for inclusion in the PDB-REDO databank. On a weekly basis the *Foldit* database is queried to see whether any new or updated *Reconstruction puzzle* results are available. If so, the associated PDB-REDO entries are marked for replacement in the next update cycle.

Whenever a *Foldit* model is used, this is clearly documented in the PDB-REDO metadata. In these cases, if the *Foldit* player has agreed to have their *Foldit* name attached to the entry, the PDB-REDO webpage will give credit to the *Foldit* player whose (redone) structure model is now available for public download. The use of a *Foldit* model in PDB-REDO entries can also be queried in the PDB-REDO archive manager at https://pdb-redo.eu/archive/ with the property FIUSED set to true. Independently, the *Foldit* players also maintain a Fandom wiki about the combined *Foldit*and PDB-REDO resource at https://foldit.fandom.com/wiki/PDB-REDO_Foldit_results.

## 4. Conclusions

With the success of the *Foldit* players in improving experimental structure models, the *Reconstruction puzzle* series within *Foldit* continues, and will continue, to provide improvements to known structures. With the success of this test, the *Reconstruction puzzle* series can be used to investigate many questions within the large knowledgebase of structural biology. At present, these puzzles are only for crystallographic structure models, but with the increase in the number of cryo-EM structure models, the same problems also persist there, and can also use the intervention of *Foldit* players.

With the focus on new artificial intelligence tools and how they can improve fields including structural biology, cases such as this are here to remind us that the greatest untapped potential for science is that of humanity.

## Supplementary Material

Supplementary Information S1: Player strategies. DOI: 10.1107/S2059798325008149/lie5003sup1.pdf

Supplementary Information S2: PDB-REDO statistics. DOI: 10.1107/S2059798325008149/lie5003sup2.xlsx

Supplementary Information S3: Full list of Foldit Players. DOI: 10.1107/S2059798325008149/lie5003sup3.pdf

## Data Availability

The *Foldit* software is available from https://fold.it. The benchmark results are available as supporting informationand the final structure models are available through https://pdb-redo.eu.
