# Supplementary material for: Reconstructing biological molecules with help from video gamers
Source: Acta Crystallogr D Struct Biol. 2025 Oct 8;81(Pt 11):598–604. doi: 10.1107/S2059798325008149 (PMC12576847; doi:10.1107/S2059798325008149)
Supplement: Supplementary file 1 [file d-81-00598-sup1.pdf]

STRUCTURAL BIOLOGY

Volume 81 (2025)

Supporting information for article:

Reconstructing biological molecules with help from video gamers

Andreas C. Petrides, Robbie P. Joosten, Foldit Players, Firas Khatib and Scott Horowitz

Supplemental Information S1 - Foldit Player ED strategies

S1.1 Glossary of in-game tools

Shake:	https://foldit.fandom.com/wiki/Shake
Wiggle:	https://foldit.fandom.com/wiki/Wiggle
WP = Wiggle power:	https://foldit.fandom.com/wiki/Wiggle_Power
Band:	https://foldit.fandom.com/wiki/Band
Voids:	https://foldit.fandom.com/wiki/Voids
Remix:	https://foldit.fandom.com/wiki/Remix
Rebuild:	https://foldit.fandom.com/wiki/Rebuild
Freeze:	https://foldit.fandom.com/wiki/Freeze
Idealize:	https://foldit.fandom.com/wiki/Idealize
CI = Clashing Importance:	https://foldit.fandom.com/wiki/Clashing_Importance
Cutpoint:	https://foldit.fandom.com/wiki/Cutpoints
Tracks:	https://foldit.fandom.com/wiki/Tracks
Auto structures:	https://foldit.fandom.com/wiki/Auto_Structures
Electron density (tool):	https://foldit.fandom.com/wiki/Electron_density_(tool)
ED = *Electron Density*:	https://foldit.fandom.com/wiki/Electron_density
SS = *Secondary Structure*:	https://foldit.fandom.com/wiki/Secondary_Structure
Trim/Trim Tool:	https://foldit.fandom.com/wiki/Trim
Pull tool:	https://foldit.fandom.com/wiki/Pull
Evolver:	https://foldit.fandom.com/wiki/Evolver

S1.2 General observations of player strategies

As mentioned in the main text, some of the players with top solutions only used automated scripts to improve their structures. While the other top players did not *solely* use scripts, it is clear that all players who succeeded in improving multiple PDB structures all heavily used automated scripts within Foldit. Every player that improved the initial structure on at least 5 different puzzles told us that they mostly used automated in-game scripts.

While there were variations in the different specific Foldit *recipes* used by these top players, and especially in the order they were run, they all used similar tactics to improve their models, highlighting again that considerable improvement in automated fitting of structural maps can be accomplished.

Automated scripts used by the top performing Foldit players

Rebuilding scripts: Attempt to replace parts of the chain with different protein fragments
Banding scripts: Create distance constraints between regions of the protein before minimization
Cut & Wiggle scripts*:* Break the protein chain into subsections before minimizing each region
Local Wiggle scripts: Perform individual minimizations on subsections of the protein chain

Additional scripts used by most of the top performing Foldit players

Quake scripts: Compress the protein using distance constraints to remove any cavities in the structure
Fuze scripts: Minimize the protein chain while alternating the penalty for steric clashes
Genetic algorithm scripts: Deform the protein using random constraints before minimization.<br>Keep only constraints that improve the score, then repeat the process.

S1.3 Analysis of the top player's strategy (with 10 top solutions)

Galaxie, the Foldit player who improved 10 of the 58 structures, used a series of recipes for all the puzzles. The way they progress through the different recipes depends on which version of *Wiggle* (gradient-based minimization) they use in the game. *Low Wiggle Power* does not force ideality computations during minimization in Foldit, making it faster, whereas the slower *High Wiggle Power* forces all ideality computations during *Wiggle*.

As noted by another player, *NinjaGreg*, in S1.4:

> "It is particularly important to do as much score improvement as possible with the wiggle power on Low in the beginning as this is when the protein is most relaxed and susceptible to movements. Once the switch to High wiggle power has been made, the protein gets "stiffer", harder to reshape."

Galaxie's strategy is consistent with the above statement, as they first start running specific recipes with *low wiggle power* before progressing to *auto wiggle power.* In *low wiggle* mode—not worrying about ideality during minimization—*Galaxie* uses *rebuilding* scripts*,* which perform *fragment insertions* in Foldit*,* and *banding* scripts which insert constraints (or rubber bands, in Foldit) between regions of the protein before minimization.

Once finished with *low wiggle power*, *Galaxie* changes their folding technique by performing the following tasks in *high wiggle power* mode: *cutting* the protein chain into subsections and performing individual minimizations, using *banding* scripts again, and finally running a *microIdealize* script—which goes through the entire protein chain, cutting it into subsegments, idealizing each one (setting all angles and bond lengths to "ideal" values) and then minimizing (example *microIdealize* recipe: https://fold.it/recipes/48411).

One of the 10 structures *Galaxie* improved on was the first Foldit Reconstruction puzzle that incorporated DNA bound to the protein—we were testing if Foldit players could improve both the protein and the DNA using the electron density. *Galaxie* explained in S1.4: "I start by looking at the structure to see how it fits in the [Electron Density cloud]. If DNA is present, I try to get the bonds in line for the correct pairs."

S1.4 Top player strategies, in their own words

NinjaGreg

1. I begin by setting the *wiggle power* to Low. This gives the segments the most flexibility to adjust their shape.

2. Since we can assume that the initial fold of the protein is pretty close to what it should be, I don't apply any hand-folding.

The first thing I do is get the protein as relaxed as possible.

3. I *shake* until it has made at least two passes over the protein. This receives a lot of the stresses that occur as part of importing the protein into Foldit.

4. I *wiggle* until the score stops changing. This also releases much of the stresses from the import.

5. I try another *shake*. If it gets points, I repeat steps 3 and 4 until no more score gain is achieved.

6. I then run the script "*cut and wiggle*", which cuts the protein every N segments, then *wiggle*s all segments. This will allow the segments to move more freely, and will release more of the stresses in the segments.

7. I then run the script "*micro idealize*", which applies a cut to one end of each segment, then "idealizes" that segment, then removes the cut and wiggles that and a few surrounding segments.

8. I then run the recipe "*QuakeR*", which uses *bands* to apply compression to selected segments, looking to "squeeze out" any avoidable voids inside the protein. It's sort of like squeezing a Brillo pad to reduce its volume.

At this point the protein is pretty relaxed, so we can start seeking to improve the configuration. If disulfide bonds need to be connected, this is the point to do it. I use hand-folding with *bands*, it's pretty fast.

9. I normally then run the script "*fracture*", selecting the "early" option, using the first option presented. I run this overnight, allowing the script to hopefully examine the entire protein at least once. This option of fracture works on the first three segments, then one more each pass up to six segments, *rebuilding* the selected segments multiple times, and using the highest scoring rebuild if it improves the overall protein score (otherwise it reverts back to the positions at the start of that set of segments.)

10. I then run the script "*AFK3*", which reduces the *clashing importance*, *wiggle*s until the score stops declining, *shake*s the protein to relax the side chains into more favorable positions, raises the *clashing importance* back to full and *wiggle*s until the score stops improving. It may repeat

this process with a smaller reduction in the *clashing importance* to see if it can improve the score still more. It repeats this process until it fails to improve the score a given number of times (or until I see it not making progress, at which point I cancel it).

11. I may then run other scripts to continue to wring points out of the protein, which means that the energy of the protein is being reduced. Typical programs I run at a this point are "*JET*", which does *wiggling* in sets of segments of various lengths to look for more points (less energy); "*fuze using cuts*", which places a *cutpoint* at selected places, does a "fuze", removes the cut, and "fuzes" again; "*TvdL enhanced DRW*", which is similar on concept to fracture, but investigates *rebuilds* based on a list of different criteria; "*nbl_hinge*", which uses a variety of sub-scripts to massage the protein; and sometimes "*Random idealize*", which randomly selects groups of segments and idealizes them, then *wiggle*s the result to see if the score is improved.

I run the various scripts until it appears that as much improvement as possible has been achieved. I then set the *Wiggle* Poser to High and repeat the above sequence.

The End Game

Once the above sequence has squeezed the protein until little more progress is made, then I resort to running first "*Banded Worm Pairs Infinite*" with *wiggle power* set to Low, then "*AFK3*" with *wiggle power* set to High. I repeat these two until they stop yielding points, then try various of the above scripts to seek further improvement, doing this until time runs out on the puzzle (usually one week).

It is particularly important to do as much score improvement as possible with the *wiggle power* on Low in the beginning as this is when the protein is most relaxed and susceptible to movements. Once the switch to High *wiggle power* has been made, the protein gets "stiffer", harder to reshape.

Galaxie

I start by looking at the structure to see how it fits in the **ED**. If DNA is present, I try to get the bonds in line for the correct pairs.
Starting with low *wiggle* and progressing to auto *wiggle,* I use a series of *rebuilders* and *band*ers. *Cut* and *wiggle*, more *band*ers and *microIdealize* are used in high *wiggle*. End game usually involves "*band*ed *worm pairs*". Sometimes *rebuilders* and "*acid tweaker*" are helpful to further refine the structure.

MicElephant

I currently have a typical sequence that I use for the smaller **ED** puzzles.

First steps all with *wiggle power* auto:
1) *Wiggle sidechains*, *shake* sidechains, *wiggle* all. Repeat this sequence until no more gains.
2) "*Tvdl Walking Rebuild*", length 1 to 5 for smaller puzzles, about 12 hours for the real big ones.
3) "*Tvdl remix"*, length 3 to 6, until no gains for ~1 hour. Sometimes I use "*Jolter 2.0"* instead.
4) "*Microidealize 4.1.1*"
5) "*Cut&wiggle everything*", length 3
6) "*Tvdl DRW compressor 2.0*", length 2 to 6, until no more gains for about an hour.
7) "*Sidechain flipper*", using extensive mode
8) "*nbl_hinge 3.4*" with default settings
9) "*Total LWS 2.0*"

Now *wiggle power* medium:
10) *Wiggle* all
11) "*AFK 3.1*"

Now *wiggle power* high:
12) *Wiggle* all
13) "*AFK 3.1*"

Now *wiggle power* low:
14) "*BandedWorm Pairs IntFilt 1.4.9"* until 5 to 10 points are gained

Back to *wiggle power* high:
15) From here on: no fixed sequence. Repeat one of the recipes mentioned above.
16) Repeat step 14 (low) and 15 (high)

Sometimes if I see bad parts of the protein which are not fixed by the recipes I do some hand-folding between the steps, e.g. to get sheets more parallel, or if a sidechain is not in the density cloud at all.

Hillbillie

First of all, my approach to the mentioned puzzle is the same that I use for all puzzles with all work done by self-programmed lua scripts. Since I have no background in protein construction or manual improvements to these structures, I simply started to use scripting to do all of my work. The main indicator in this working process is the total score after different types of interactions with the structure and using most of the capabilities of the given scripting commands.

I'm still trying to improve my results by adjusting my scripts and letting them do their work automatically as long and as fast as possible. The choice of which kind of interaction to be done is mainly based on random selection.

So you can say my results are based on lucky guessing and finding the best possible ways of improvement.

This might not sound very groundbreaking, but that's my way of working through the puzzles and trying to create powerful algorithms. I hope I could give you a brief look at my work.

Bruno Kestemont

The good news is that for these revisited puzzles, I don't hand-fold nor do I show the **ED** map: everything is done with recipes. This suggests that everything could be automated for mass treatment.

I will start by explaining my overall strategy, then I will document what I did for the specific puzzles.

Overall strategy
I always set *wiggle power* to low. When the deadline is <3 days, I turn to high *wiggle power*.

I usually start 3-5 different *tracks* on the same puzzle. It depends on the available memory.
I start with "*Auto structure*".
On different *tracks*, I run *shake*-*wiggle sidechains*. One track with *wiggle sidechains*-*shake*- *wiggle sidechains*.

Then I start with a different recipe on each *track*, overnight.

I always use the following 2 recipes within the period of the first 4 days on low WP:

- "*jolter 2.4*" (**J** for short), days 1 to 3, 8-24 hours running. I can stop it after 12 hours, run auto-structure and start it again, until there is no more gain.

When hand-folding on previous **ED** puzzles from scratch, I observed that secondary structures that were already roughly hand positioned in a cloud would then be finely positioned with full (helix/sheet) *rebuild* and in particular this **J** recipe. Thus, this is a perfect recipe for refining **ED** puzzle solutions. Also, the loops find a reasonable position in the cloud with this (if there is no time left and the helices and sheets are already well positioned, I could focus the recipe on loops only). The recipe *rebuilds* all identified SS and loops infinitely in turn.

- Cut and *Wiggle* Random v1.1 (**cwr** for short), day 1 to 4 (preferably after other recipes because it tends to stick the protein), 8-24 hours running, until it gains less than several points per loop.

When hand-folding on previous **ED** puzzles from scratch, I observed that a rough hand positioning of a *cut point* in the cloud "automatically" perfectly fitted the section in the cloud using *wiggle all*. Thus, I'd cut a section, put in the cloud, and *wiggle all* that section. I positioned the helices and sheets piece after piece just like this. When I was finished, I closed the *cutpoints* before using *wiggle* again.
Thus, *Cut and Wiggle Random* (**cwr**) is an excellent recipe for fine tuning an already solved **ED** puzzle.
Note that this gives me an idea for a new recipe combining "*Jolter*" and "*Cut & wiggle*" (cutting before and after a SS before to *wiggle*, then uncut). Thus cut & *wiggle* SS (not random). To code asap.

If there is time left, I then run the following in order to *rebuild* low density score segments.

- Fracture v2.1 w/ *Remix* (**fr** or **frw** for short). Day 2-4, 8-16 hours.

The options are set as follows: "*Early game - Fast & Loose*", "*I'll have banders with that*", "*Do remix instead of rebuild ...*" (if there is enough time left), and certainly "*Target density subscore*".

If there is time left, I could run a recipe including a random *idealize* function, like:

- *Quaking Rebuild V2 1.0 RI* (**qrri** for short)

The other recipes I could run as first recipe before **J** and **cwr** are:

- AFK3.5.1 (BounceWiggle) (**afk** for short with no *shake,* or **afks** for short with *shake*)
- Quickfix 3.6.3 + faster cleanup (**qf** for short)

The second recipe (day 2-4) is always **J**, the third recipe is always **cwr**.
The fourth recipe is **fr** if there is time left.

Then if there is time left, I run a *local wiggle* recipe in order to refine before switching to high *wiggle power*:

- JET 4.2.7 (**jet** for short)

Other recipe I could use on day 3-4 before *JET 4.2.7* if there is time:

- Ebola 4.3 (option velocity 5) (it rebuilds the worst long portions)

Then, switch to high *wiggle power* and run the following recipes:

- nbl_hinge_v4.2.2 (**nblh** for short)
- *Banded* Worm Pairs Inf Filt 3.5.7 (**bwpi** for short)

Specific puzzles

(Puzzle# 2224)
Auto structure, *shake*, *wiggle sidechains*
On low *wiggle power*: **afks** + **fr** +**J** + **cwr**
On high *wiggle power*: high **nblh** + auto **bwpi** then several recipes in high *WP* in parallel with auto **bwpi**

(in parallel means that several *tracks* run the same solution in parallel, saving and loading regularly from local share in order to start over with the best one on all *tracks*).

(Puzzle# 2300)
It was a small puzzle, so I was able to use my full range of favorite **ED** recipes.
Auto structure, *shake*, *wiggle sidechains*
On low *WP*: **J** + **cwr** + **J** + **qf** + **qrri** + **fr** + **ebola** + **cwr** + **jet**
On high *WP*: high **nblh** + auto **bwpi** then several recipes in high *WP* in parallel with auto **bwpi**

(Puzzle# 2359)
Auto structure, change segments to helix and idealize this portion, *shake*, *wiggle sidechains*
On low *WP*: (probably **cwr** +**J**+ **fr**) + **ebola**
On high *WP*: high **nblh** + auto **bwpi** then several recipes in high *WP* in parallel with auto **bwpi**

(Puzzle# 2368)
Auto structure, *shake*, *wiggle sidechains* (or reverse, I don't remember)
On low *WP*: (probably j+ **cwr** + **fr**) + **qrri** + **ebola** + **cwr** + **jet**
On high *WP*: high **nblh** + auto **bwpi** then several recipes in high *WP* in parallel with auto **bwpi**

---

grogar7

I start by doing a whole-protein *shake* and *wiggle* at *CI* =0.2 to start. Then, with these larger proteins, I trim to the worst scoring areas and *rebuild*. Then I revert to the standard techniques I have described in other communications with you.

Spmm

My general approach: try to identify any large hydrophobics, helices and the ‘ends’ in the density cloud, attempt to understand where the ‘folded mass’ is located and assume I will need to start again once a picture emerges.

---

gmn

No problem sharing my approach. It hasn't changed much if I'm using the *trim tool*.
Approach depends on the size of the protein.

Very large ED proteins (multiple subunits):

Start with CI 1.0/Low *wiggle*
Shake/*wiggle*
Fix any bad scoring areas with *remix* (usually the red segments) or rebuild if *remix* doesn't work
Freeze entire protein
Unfreeze only the subunit I want to work on and highlight it for *trim tool*
Rebuild trimmed subunit with scripts
Untrim/*shake*/*wiggle*
Unfreeze entire protein
Shake/*Wiggle* (compare to untrimmed shake/wiggle score and take higher score for next subunit)
Freeze entire protein/*unfreeze* subunit I *rebuilt*/highlight for *trim tool*/run "*GAB script*" (*GAB*) on trimmed subunit
Untrim/*shake*/*wiggle*
Unfreeze entire protein/*shake*/*wiggle* (compare to s/w *untrimmed* score and take higher score for next subunit)
Repeat process for each subsequent subunit I want to work on
Run end game scripts on entire protein, which includes switching to high *wiggle* at some point, once I have completed working on all submits

Smaller ED proteins (few or no subunits):
Start at 0.5 *CI*/Low *wiggle*
Shake/*wiggle*
Fix any bad scoring areas with *remix* (usually the red segments) or rebuild if *remix* doesn't work
Freeze entire protein
Unfreeze only the section I want to work on and highlight it for trim tool
Rebuild trimmed area with scripts
Untrim/*shake*/*wiggle*
Unfreeze entire protein
Shake/*Wiggle* (compare to untrimmed s/w score and take higher score for next section)
Repeat process for each section I need to work on (Note: I do not usually *GAB* at *CI* 0.5.)
Repeat the entire process for *CI* 1.0 (see above)

If the **ED** puzzle is sufficiently small, I will start at *CI* 0.2 and run the *CI* 0.5 process using *CI* 0.2 (no *GAB*). I will then follow up with the *CI* 0.5 process and add *GAB* for each section (which then leads to the *CI* 1.0 process).

LociOiling

I use the same general approach on all electron density puzzles. My approach involves running different sequences of recipes on two or more parallel *tracks*.

Many of the recipes are private (or group) recipes, but all are based on public recipes.

The initial steps are always done on low *wiggle power*. I shift to high *wiggle power* later, roughly halfway through the week that the puzzle runs.

The first step is to "*wiggle* out" the starting pose. I first *shake* (keyboard hotkey: S), then *wiggle sidechains* (hotkey: E), then *wiggle* all (hotkey: W). I usually let the *wiggle* steps go until the counter spins. I repeat the S/E/W action two or three times.

At this point, I "nudge" the protein by *pulling* on a segment to make the score drop. I *wiggle* again to see if the score recovers. I repeat S/E/W if it reaches a higher score. I may also use a *band* plus *wiggle* to drop the score, then delete the *band* to see what happens.

After the score reaches a plateau, I run a *Fuze* recipe, *Fuzes 3.0.3* (*Fuzes*) usually using the default settings.

After *Fuzes*, I have the starting point that I'll use on the rest of the puzzle. I usually take a look at the PDB at this point, to see if I can match the protein.

Taking the starting point solution, I launch two or three clients. Each client runs in its own directory, minimizing any interaction.

Still on low power, one client runs TvdL Enhanced DRW 3.1.1 (*EDRW*), which uses the *rebuild* function. I generally increase the number of *rebuilds* per section, from the default of 15. For a small puzzle, I would go with 100 *rebuilds* per section, but I set a lower number for monster puzzles. The number of cycles still defaults for 40, but I always reduce this to eight or less based on the size of the puzzle.

A second client runs a private *remix* recipe based on TvdL D*Remix*W 3.1.2.

I may also try a third client, using a slight modification of the public *BandFuze* recipe. This recipe keeps running until cancelled. Sometimes I may *remix* or *rebuild* the results.

After the *remix* completes, I switch to *rebuild*, and *rebuild* switches to *remix*. With the trend toward very large puzzles, I may reduce the number of cycles at this stage.

Once *remix*ing/*rebuilding* is complete, I switch to high *wiggle power*. The exact timing of course depends on the size of the puzzle. Usually the *rebuild*/*remix* and *remix*/*rebuild* solutions are still in the running at this point. The “*BandFuze*” solution may also still be viable.
The first recipe on high power is a private "*GAB*" *band*ing recipe. I adjust the number of *GAB* generations based on the puzzle size.

After *GAB*, I run a “*Cut & Wiggle”* recipe followed by a “*Microidealize”* recipe. My versions are more persistent than most of the public versions.

After "*Cut & Wiggle*" and "*Microidealize*", I switch to an "*LWS*" recipe, still on high *wiggle power*. I use a private recipe that's based on "*Band Worm Pairs*" to start.

When my private recipe begins to plateau, I usually switch to auto *wiggle power*, and run *Banded Worm Pairs Inf Filt 3.4.10* (BWP IF) by Bruno Kestemont.

At this late stage, I also assess the competition. I may continue with an *LWS* recipe if I'm in the lead. Sometimes, corrective action may be helpful, such as additional *remix*ing or *rebuilding* on high *wiggle power*. An "*Acid Tweaker*" recipe can sometimes help. There are also a number of other *LWS* recipes on the bench, and sometimes they can find a point or two.

I'm working on improving *EDRW* and other key recipes to make them "chain aware", since many of the recent **ED** puzzles have two or more chains. I'm guessing that *remix*ing across chain ends is never a good idea, but it's not clear how much time it actually wastes. A similar argument applies to *rebuild* and other actions.

On one of the puzzles, an "*Acid Tweeker*" near the end found a large gain. Otherwise, the process seemed to be much as described.

After running "*Band Worm Pairs*" on auto for a while, I may switch back to high *wiggle power* if time permits. Going back to high WP often finds a few points.

I've tried the *trim tool* on several puzzles with mixed results. On puzzle 2439, I tried *remix*ing and *rebuilding* adjacent pairs of chains. This still took forever due to the large number of chains. The score was lower after finishing each pair. The winning solution was based on tackling the entire protein. On other puzzles, working on trimmed sections separately has produced gains instead of losses. I think the "everything all at once" strategy was still the winner in most of these cases. The *trim tool* itself seems to be working well, so possibly a better strategy is needed. Currently, I've been *shaking*, *shaking*, *wiggling* and *fuzing* after *un-trimming* each section. Maybe saving these steps until all sections have been processed would work better.

---

toshiue

I found my notes on that puzzle and as I had expected, there wasn't anything unusual (for me) in the approach to that protein. That said, I don't have visibility on the approaches taken by others, only those that I've done on **ED**s in the past. You have a greater sampling to compare to, and my notes are four pages, if you'd like a copy.

I/we bump up against and sometimes exceed the theoretical limits of a protein. I think puzzle 2300 was just one of those instances where more things went right than usual. I don't have a rigid approach to folding. I primarily do *Evolver* only with enough soloist-folding to get a taste for the protein.

To me, each protein is an individual and I approach them as such. Group dynamics plays a greater role than one might imagine. When most of our group veterans are present and we're slightly giddy, we are truly at our best as a group. I returned to folding when I noticed the *Electron Density Reconstruction series* (EDR) had continued (Puzzle 2300 being one of the EDR series). These puzzles are very easy for me to work with and I thoroughly enjoy them. Thus, the hours get put in for these ones. On that note, putting in the hours is a necessity. Hardware matters more than most seem to believe. Iterations matter.

I run clients on all three major OS: Windows, Mac, Linux. Recipes respond differently to each. Often when we push a protein hard on one of our favorite (demanding) recipes, we get swarms of crashes. Switching over to another OS very often cures this. One last item, on the EDR series. They're too anonymous. Descriptors such as the one on EDR88 don't entice us into all-nighters. Adding real world descriptors (ie... It's a delivery system for a new pediatric cancer drug at Stanford Medical), and we're pulling all-nighters.

Sorry I didn't have a magic algorithm to offer, but these proteins really are individuals. We have to get to know them before we know what we can do with them. Also, I think too many players are overly cautious. There's a cultural norm as to approaches with the folding regimen that everyone seems to strive for. I routinely break with those norms.

AlphaFold2

Just a few simple tips on Electron Density Reconstruction Puzzles.

Before I start to use any tools, I will rotate the protein 360 degrees to see if there are any areas that stand out from the rest. Patterns: I identify these by looking for amino acids that are not fully extended or hydrophilic or hydrophobic and any segment of the backbone that may have a better potential in a new configuration.

Choosing the right place to start is always tricky, so rotating the protein allows you to see the full 3D shape you have existing, the colour of the segments are ideal when they are green, if the backbone colour is brown, this area can be rebuilt to find a new position that gives a better score "or" lower score initially, but the protein sits in a better position, by *shaking* and *wiggling* the protein will mean you can see how it's movement and new *rebuild* holds together or just the area you *rebuilt*.

Modifying approximately 3 at a time will give you an indicator if you are working on the right spot.

Sometimes having the amino acids in a different position and rebuilding those segments can help find new positions that weren't available in the existing position. As the backbone is changed, it gives the possibility to find new energy levels that reflect the bonds to the surrounding amino

acids within the existing structure. Some attract, some repulse, and others are neutral to one another. Once the backbone has settled, you will be able to identify any areas that have changed from brown to green or vice versa, thus working on connected areas (segments) around the protein that “look” like they could be in a different combination can be tried.

Often when an amino acid or more are out of place, you can see that it’s a brownish colour, and by manipulating the backbone into a new position will drop the energy level but once you have *Shaked* or *Wiggled* it will now take into consideration the new positions. Then by reducing the *clashing importance* lower you can make the backbone relax, and then adjusting the *clashing importance* higher will give you some movement and it will explore the new environment you have given the protein to *wiggle* out.

With the Electron Density Reconstruction puzzles you will possibly get some areas that have a exploration area that are ghosted over top of the existing protein. You can try and *rebuild* the backbone to fit into this exploration area and after each adjustment, *shake* and *wiggle* out the protein to see how each little movement affects the score and shape of the protein.

If you try and make adjustments BEFORE *wiggling* the protein out, *rebuild* a few segments first, then decide whether to *shake* and or *wiggle* because the protein is usually hard to move much for these puzzles.

Some of the amino acids will give you an idea of where in the protein structure they prefer to be: start or end of helix or the sheet. As you get familiar with different amino acids and the backbone structure—along with segment colours—you will be able to choose a better configuration to hunt for a better potential and find a shape that’s more natural.

---

blazegeek

I've been struggling to find a way to describe my approach as I don't really have one. The only consistent pattern I notice is that I try several different approaches simultaneously until there is an obvious score leader. I then try several approaches using that solution until, again, there is a clear leader.

I will point out that I don't have a background in molecular biology or related fields (I actually studied television production). In fact, I still have a hard time remembering the 20 amino acids without forgetting one or two. I suspect that my lack of knowledge and understanding enables me to try things that most others wouldn't consider trying, resulting in a sort of "beginner's luck" on occasion.

Being neurologically atypical is certainly a factor in my approach to problem-solving of any kind, but especially within the highly stimulating, interactive environment of Foldit. Unfortunately, it would be impossible to describe what that process entails. I tend to look at a protein aesthetically;

some shapes are much more pleasing than others, more beautiful, more elegant. It's useful to be able to visualize this way, as that elegance seems to exist throughout Nature and at all scales. It may not be very scientific, but one doesn't need to understand something to find it beautiful. That, too, is rather elegant in itself.

I don't know how helpful this is for your paper, but I appreciate the opportunity to share my approach. In a nutshell, I tend to approach these puzzles quite abstractly, which occasionally pays off. I'm happy that something useful came of it!
