# Supplementary material for: Reconstructing biological molecules with help from video gamers
Source: Acta Crystallogr D Struct Biol. 2025 Oct 8;81(Pt 11):598–604. doi: 10.1107/S2059798325008149 (PMC12576847; doi:10.1107/S2059798325008149)
Supplement: Supplementary file 3 [file d-81-00598-sup3.pdf]

STRUCTURAL BIOLOGY

Volume 81 (2025)

Supporting information for article:

Reconstructing biological molecules with help from video gamers

Andreas C. Petrides, Robbie P. Joosten, Firas Khatib and Scott Horowitz

Supplemental Information S3 - Foldit Players

Alan Coral

Bruno Kestemont

Deniz Akçay

Douglas Wheeler

Gary Morrison-Nelson

Gary O. Gross

Gil Beecher

Gregory T. Hansen

Keith T. Clayton

Linda J. Wei

Mathias Flack

Michael Simon

Sándor M. Szilágyi (George Emil Palade University of Medicine, Pharmacy, Science and Technology of Targu Mures)

Susan P. Martin

Sven M. Holst

Walter Barmettler
